# Who Shot the Bullet? Projectile Composition Characterization as an Evolutionary Method for Enhancement of Ballistics Evidence Analysis

**DOI:** 10.1021/acsomega.3c06316

**Published:** 2024-01-08

**Authors:** Ashley Newland, Emilynn Banks, Jan Halámek

**Affiliations:** † Department of Environmental Toxicology, Texas Tech University, 1207 Gilbert Drive, Lubbock, Texas 79416, United States; ‡ The Institute for Forensic Science, Department of Environmental Toxicology, Texas Tech University, 1207 Gilbert Drive, Lubbock, Texas 79416, United States

## Abstract

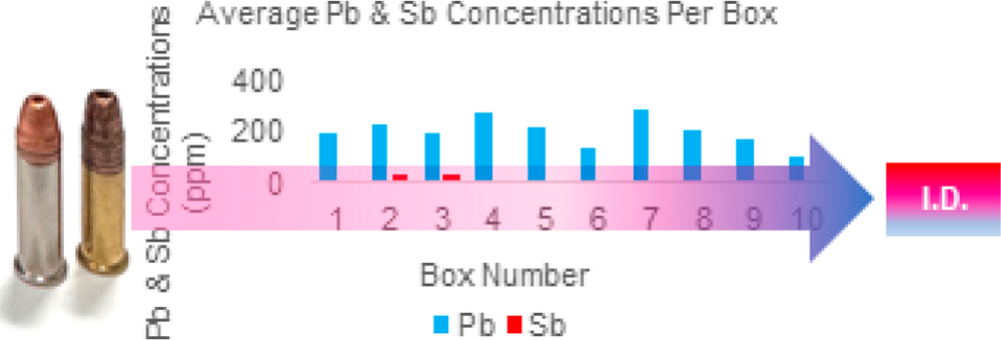

Toolmark and Firearm examiners’ opinions have fallen under scrutiny as inadmissible ballistics evidence has led to the possibility of wrongful convictions and cold cases that could have been solved with the presence of a physical bullet, casing, and/or weapon at the crime scene. This research provides a solution for subjective-based conclusions and the absence of physical evidence altogether. Analysis of bullet material using Atomic Absorption Spectroscopy (AAS) has distinguished bullet composition between manufacturers from a surface scratch. This provides proof of concept that, when a bullet strikes a surface, metal deposits can be extracted and analyzed to corroborate microscopy techniques that currently violate Daubert criteria. Further studies could also provide results to distinguish barrel manufacturers from fired bullets and casings. This novel method of analysis can pave the way for crime scene collection procedures in the absence of physical evidence and provide an increase in scientific value to the expert’s conclusions.

## 1 Introduction

Ballistics analysis is an important aspect of crime scene investigation due to the frequency of illegal firearm use. The National Incident-Based Reporting System (NIBRS) reported that 2012–2021 offenses of illegal weapon use contained over 1 million offenses observed by at least 11,000 law enforcement agencies. This data comprises 64% of all criminal offenses, which highlights the need for enhanced ballistics analysis.^1^ Uniform crime report data provided by the Federal Bureau of Investigations states that approximately 340,000 homicide cases are unsolved as of 2022 and it is predicted that 4–6% of incarcerated individuals may be wrongfully convicted with a majority of those cases involving violent firearm use.^2,3^ The rate of ballistics-involved crimes demands that new methods of analysis must be developed to increase validity of results, reduce the number of wrongful convictions, and prevent future cold cases involving firearm use.

As current methods of ballistics analysis have been used for nearly a century, a vast number of peer reviewed publications and research has shown that comparison microscopy is the only method in which all ballistics analysis is conducted when bullets or casings are found on scene.^4,5^ Due to diverse experience of ballistics examiners, the error rates and standards by which conclusions are reached with this technique fall under legal and scientific scrutiny. Acceptance of these conclusions and practices within the field are therefore decreasing and wrongful convictions may be observed. It is pertinent that scientific value is added to microscopic examination of toolmark comparison as observation alone does not yield accurate, precise, or repeatable results when experts have the ability to reach different subjective conclusions based on their own experience and qualifications. Due to this subjectivity, current techniques have the potential to be unreliable and inadmissible in court according to Daubert criteria. Requirements of admissibility from Daubert standards 1 and 2 state that methods have been tested and subjected to peer review and publication, which comparison microscopy techniques satisfy. Criteria 3 and 4 require known error rates and acceptance throughout the scientific community.^6^ Current ballistics analysis does not fully satisfy standards 3 and 4 due to subjective opinion.

In June of 2023, the Supreme Court ruled that the firearm examiner on the case was unqualified to reach the conclusion that the murder suspect’s firearm was a match to the fired bullets found on scene in Kobina Ebo Abruquah v. State of Maryland. Following a conviction in 2013 by ballistic analysis, the judge in this proceeding stated that firearm identification has not been shown to be reliable and the majority of the juror candidates do not question the reliability of ballistics examination.^7^ Marquette Tibbs v. United States is another landmark case in which ballistics evidence was determined to be inadmissible due to unreliable principles, methods, and peer review.^8^ These cases serve as examples for the necessity of increased scientific value in firearm and toolmark examinations as the source of the bullet was potentially misidentified, as well as current legal trends in the admittance of subjective ballistic evidence.

The aim of this research is to evaluate the efficacy of AAS as a method for analysis of elemental composition of bullets and introduce a solution for instances where ballistics evidence can be evaluated when casings, projectiles, and weapons are not found on scene. AAS has historically served as an acceptable and straightforward method for metal detection and quantification. This project provides proof of concept that a database of elemental ratios from every manufacturer and bullet type for comparison to quickly identify the origin of ballistic evidence is possible to develop, thus improving upon current standards and techniques for forensic analysis of these evidence types. It was found that AAS can produce results that distinguish bullet manufacturers from one another based on lead to antimony ratios of unjacketed bullets that were determined to be specific for each brand tested.

Atomic absorption spectroscopy was chosen as the method for this study due to its sensitivity, precision, low limits of detection in parts per billion, and ability to produce results within minutes. In comparison to Inductively Coupled Plasma Mass Spectrometry (ICP-MS), AAS is more affordable and accessible to laboratories that do not have funding for the ICP-MS instrument. ICP-MS is similarly an elemental detection method and can detect parts per billion levels. However, for ballistic evidence analysis, parts per million is sufficient to distinguish between bullet manufacturers, as shown in this study. AAS analyzes elements individually and is suitable for the rapid analysis of ballistic components as only two elemental analyses are required to produce distinguishable results. ICP-MS also has the ability to analyze different elements at once; however, this feature is not necessary for these analyses.^9^

**Figure 1:**
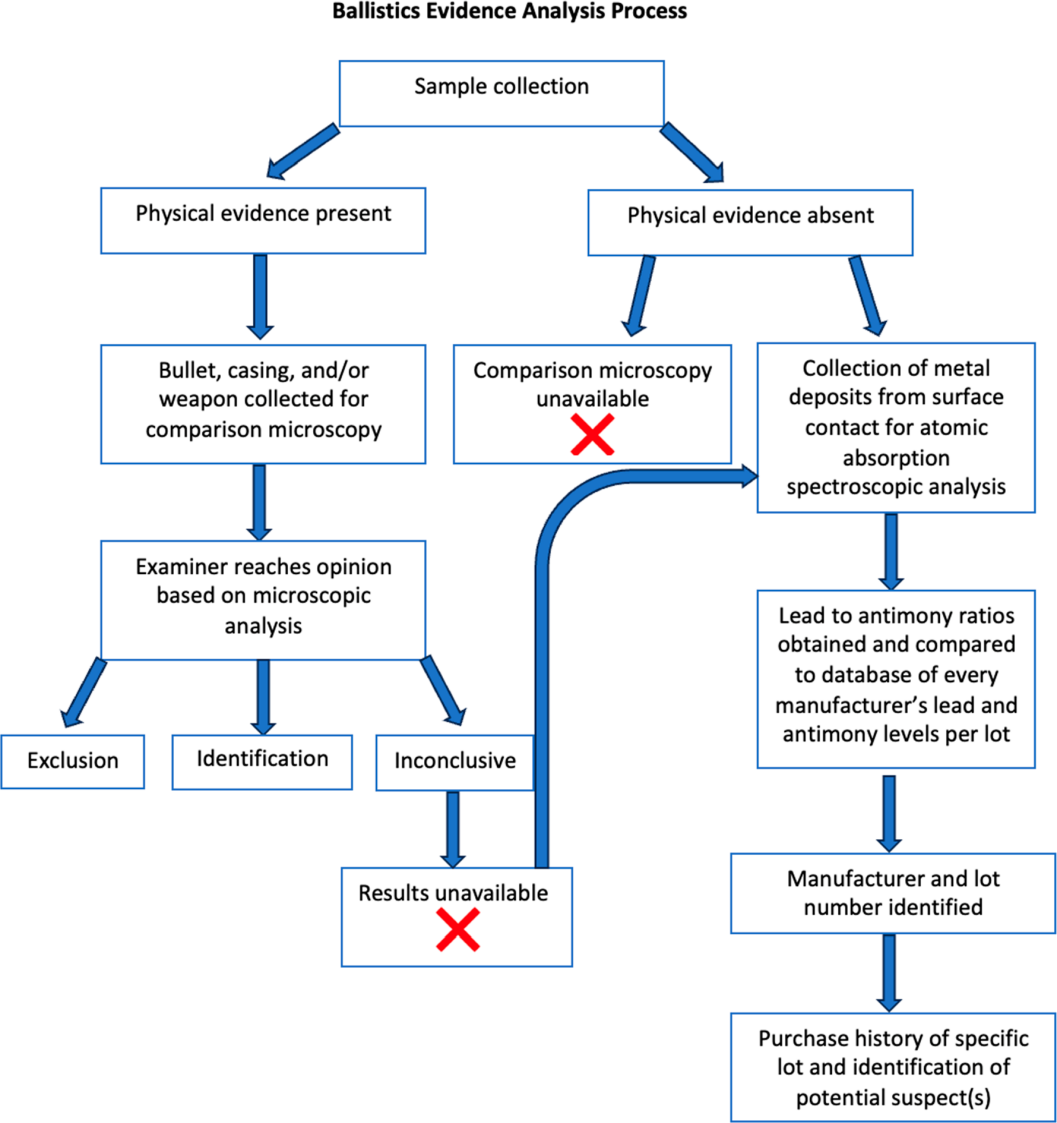
Ballistics evidence analysis process: All steps showing the collection of evidence to suspect identification from AAS and all possible conclusions from comparison microscopy.^5^

In the absence of physical bullet casings or weapon at the crime scene, ballistics analysis is significantly limited due to the need of this evidence for comparison microscopy. The most significant aspect of this research is not only to observe how effective AAS is to identify elemental composition for bullet comparison, but to introduce a method of collection for crime scene investigators in the absence of ballistics evidence altogether. Bullet impact on a surface will deposit metal from a bullet that can be collected and evaluated with AAS instrumentation. From this, it is also possible to repeatably produce a match or elimination of a specific brand of bullet to determine the firearm used in the crime and create a reference database when the surface deposits are analyzed with AAS. This approach in treatment of ballistic evidence, has the potential to revolutionize and enhance ammunition analysis, develop collection procedures, and provide scientific and statistical evidence in the courtroom that is currently unsatisfied in the Daubert evidentiary requirements. Figure 1 shows the process by which the proposed method can be implemented when physical bullets, casings, and weapons are not available for collection. Figure 1 also highlights the extensivity of information obtained compared to comparison microscopy and the ability to use AAS to solve inconclusive results from microscopic evaluation.

## 2 Materials and Methods

### 2.1 Materials

Five unfired bullets and five sample repeats equivalent to 25 samples per box of 0.22 LR caliber ammunition were analyzed. RWS (40 GR), BPR (37 GR), Armscor Precision (36 GR), Aguila Original (40 GR), Aguila Rifle Match (40 GR), Aguila Super Caliber (20 GR), CCI Stinger (32 GR), Federal Premium (40 GR), La Norma USA (40 GR), and HVHP Sellier & Bellot (38 GR) were the brands tested in this experiment. Sample preparation materials include 18.2 MΩ·cm filtered water from the Barnstead Nano-Pure Thermo-Scientific purification system, 70% isopropyl alcohol purchased fromSigma-Aldrich, 70% nitric acid purchased from Thermo-Scientific, and Diablo 100 grit sandpaper. CPI International lead and antimony standards were used to generate a 6-point calibration curve.

### 2.2 Sample Preparation

Each bullet was removed from the casing with pliers and the surface was washed with 18.2 MΩ·cm filtered water and isopropyl alcohol to remove metal contamination. The surface of the bullet was removed as an extra precaution due to contact with the pliers. This was completed by scratching off the surface of the bullet using 100 grit sandpaper on each side. Once these preventative measures were completed, the bullet was scratched against 100 grit sandpaper in five locations for repeated sample collection to determine accuracy and precision of analysis. Different bullets from the same brand were scratched on different pieces of sandpaper along with bullets of different brands. Once approximately 1 mg of metal deposits were produced on the sandpaper, a disposable plastic 1 mL pipet was used to scrape the metal into a 10 mL beaker. Metal deposits collected from each 10 mL beaker was mixed with 1 mL of 70% nitric acid. To allow for the sample to dissolve thoroughly, the metal remained in solution for 30 min until homogeneous and was diluted to 3% with 18.2 MΩ·cm filtered water.

### 2.3 Instrumentation

Thermo Fisher Scientific AAS Atomic Absorption Spectrometer iCE 3400 AAS with flame atomic absorption spectroscopic (FAAS) capability was used in this study. CPI International lead and antimony cathode lamps were used as the light source to detect the various ratios of these elements. Thermo Scientific SOLAAR software was used to generate calibration curves and data. The wavelength parameters were set at 217.0 nm for lead and 217.6 nm for antimony. Next, lead standards of 0.5, 1, 4, 6, and 8 ppm were prepared to generate a calibration curve. The same standard concentration and process was used for antimony with the addition of a 2 ppm standard, which replaced 1 ppm, and a 25 ppm standard for a 6-point calibration curve greater than or equal to 0.995.

### 2.4 Data Analysis

Statistical evaluation using ANOVA two-factor replication in Microsoft Excel 16.61 was conducted to determine if values obtained reflect the most accurate representation of each box tested given only 5 bullets were chosen per brand. This was completed by computation of each bullet average per box to generate *p*-values indicative of significance or insignificance between brand distinguishability. Tukey–Kramer analysis was used as a comparative test between brands to determine the honestly significant difference (HSD) for evaluation of how different each lead to antimony ratio was between all brands.

### 2.5 Summary of Methods

The interior of unjacketed bullets from ten brands of bullets were analyzed according to brand and type of bullet belonging to the same company (5 replicates). This allows for comparison of the alloy from the same type of bullet from the same brand along with different bullets of the same brand. Then, the overall comparison between each type and different brand of bullet was evaluated with ANOVA. From these data, variations between each brand were accounted for, and the ratios of elements specific to certain companies were established.

## 3 Results

**Figure 2:**
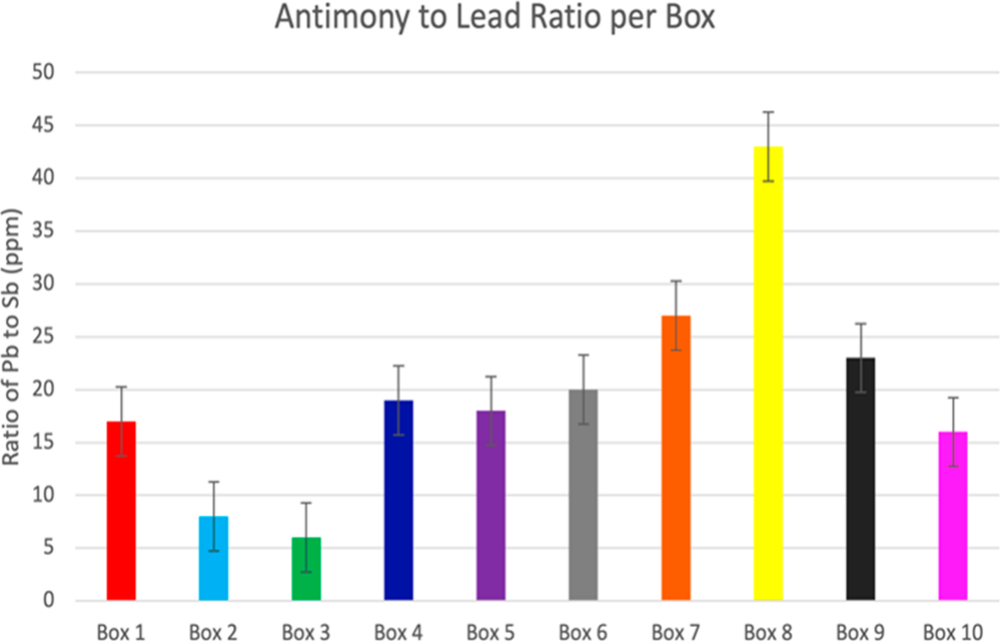
Antimony to lead ratio per box: RWS (16:1), BPR (8:1), Armscor Precision (6:1), Aguila Original (19:1), CCI Stinger (17:1), Aguila Super Caliber (20:1), Aguila Rifle Match (27:1), Federal Premium (43:1), La Norma USA (22:1), and HVHP Seller & Bellot (16:1), respectively.

**Figure 3:**
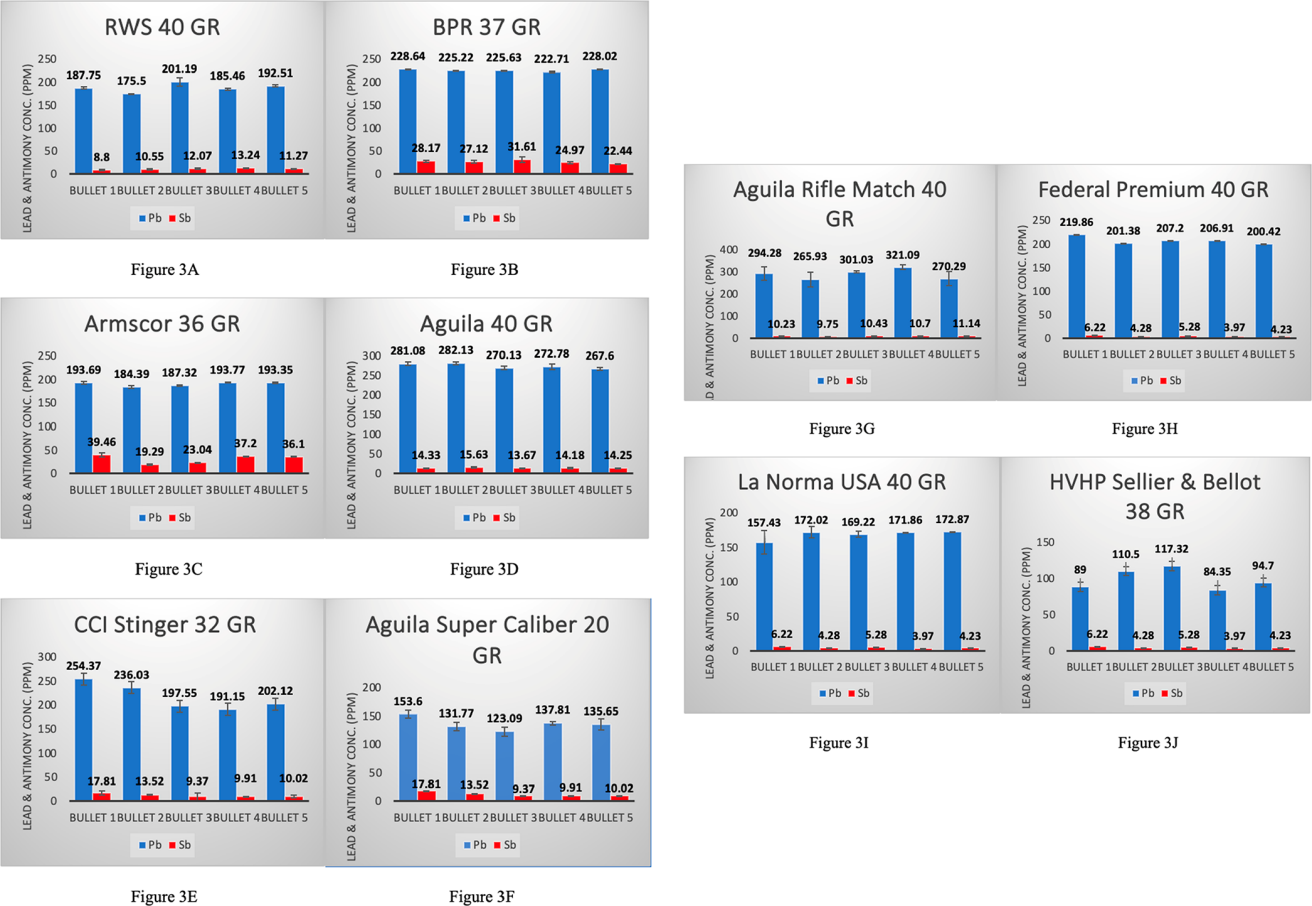
(A–J) Averages of Pb and Sb concentrations of 5 bullet repeats per box: Analysis of 5 bullet samples with 5 sample repeats from same box and 10 different brands.

Lead concentrations were higher than antimony in every sample. These numbers were expected due to the abundance of lead known to be present in metal alloys compared to other elements.^7^ For example, box 1 (RWS) lead concentration of 25 samples range from 185 to 216 ppm and antimony concentration was 10–13 ppm. Box 2 (BPR) lead concentration was 220–242 ppm and antimony concentration was 19–52 ppm. Box 3 (Armscor Precision) lead concentration is 174–202 ppm and antimony concentration was 15–52 ppm. Box 4 (Aguila) lead concentration was 254–299 ppm and antimony concentration was 11–21 ppm. Box 5 (CCI Stinger) lead concentration was 179–261 ppm and antimony concentration was 7–22 ppm. Box 6 (Aguila super caliber) lead concentration was 108–165 ppm and antimony concentration was 5–8 ppm. Box 7 (Aguila rifle match) lead concentration was 182–348 ppm and antimony concentration was 8–11 ppm. Box 8 (Federal Premium) lead concentration was 195–224 ppm and antimony concentration was 3–6 ppm. Box 9 (La Norma USA) lead concentration was 145–209 ppm and antimony concentration was 5–9 ppm. Box 10 (Sellier & Bellot) lead concentration was 83–140 ppm and antimony was 4–7 ppm. All values are shown in Table 1 to summarize all lead and antimony concentrations. Figure 2 shows comparison of the average values for all boxes. Figure 3A–J are included to highlight the abundance of lead versus antimony and illustrates the differences in concentration between each manufacturer.

**Table 1:** Lead and Antimony Concentration Ranges Per Box (ppm): Summary of All Concentration Ranges between Each Brand

lead and antimony concentration ranges per box (ppm)										
box #	box 1	box 2	box 3	box 4	box 5	box 6	box 7	box 8	box 9	box 10
Pb conc.	185–216	220–242	174–202	254–299	179–261	108–165	182–348	195–244	145–209	83–140
Sb conc.	10–13	19–52	15–52	11–21	7–22	5–8	8–11	3–6	5–9	4–7

Tables 2 and 3 include RSD% to reflect variation from bullets analyzed per box and all sample repeats. RSD values of boxes 1–10 lead analysis (refer to Table 2.0) are as follows: RWS (box 1) 6.4%, BPR (box 2) 3.2%, Armscor Precision (box 3) 3.2%, Aguila Original (box 4) 4.0%, CCI Stinger (box 5) 12.4%, Aguila Super Caliber (box 6) 13.0%, Aguila Rifle Match (box 7) 19.0%, Federal Premium (box 8) 4.7%, La Norma USA (box 9) 11.0%, and HVHP Seller & Bellot (box 10) 15.4%. Percentage of variation within each box satisfies the requirement of a less than 20% RSD value that shows precision and reproducibility of data. RSD values of boxes 1–10 antimony analysis (refer to Table 2.1) are as follows: RWS (box 1) 14.7%, BPR (box 2) 26.7%, Armscor Precision (box 3) 30.5%, Aguila Original (box 4) 15.9%, CCI Stinger (box 5) 33.4%, Aguila Super Caliber (box 6) 9.6%, Aguila Rifle Match (box 7) 9.7%, Federal Premium (box 8) 18.1%, La Norma USA (box 9) 16.9%, and HVHP Sellier & Bellot (box 10) 20.4%. These data show precision in boxes 1, 4, 6, 7, 8, and 9, whereas boxes 2, 3, 5, and 10 exceed RSD values of 20%.

**Table 2:** Lead Analysis (ppm): Average of Each Bullet Is Shown along with the Final Average Used for Ratio Determination^a^

lead analysis (ppm)										
box #	box 1	box 2	box 3	box 4	box 5	box 6	box 7	box 8	box 9	box 10
bullet 1	188	229	194	281	254	154	294	220	157	89
bullet 2	175	225	184	282	236	132	266	201	172	110
bullet 3	201	225	187	270	198	123	301	207	169	117
bullet 4	185	222	194	273	191	137	321	206	172	84
bullet 5	192	228	193	268	202	136	270	200	172	95
avg	188	226	190	273	216	132	290	207	169	99
RSD %	6.4	3.2	3.2	4.0	12.4	13.0	19.0	4.7	11.0	15.4

a RSD values are included to show variation. Results are in ppm concentration.

**Table 3:** Antimony Analysis (ppm): Average of Each Bullet Is Shown along with the Final Average Used for Ratio Determination^a^

antimony analysis (ppm)										
box #	box 1	box 2	box 3	box 4	box 5	box 6	box 7	box 8	box 9	box 10
bullet 1	9	28	39	14	18	6	10	6	9	5
bullet 2	11	27	19	16	14	6	10	4	7	5
bullet 3	12	32	23	14	9	7	10	5	7	6
bullet 4	13	25	37	14	10	7	11	4	7	7
bullet 5	11	22	36	14	10	8	11	4	6	7
avg	11	27	30	14	11	6	10	5	7	6
RSD %	14.7	26.7	30.5	15.9	33.4	9.6	9.7	18.1	16.9	20.4

a RSD values are included to show variation. Results are in ppm concentration.

ANOVA with two-factor replication was conducted on each box to show the variation between bullets, the 5 measurement repeats, and any differences of values overall. The total variance per box reflects the sum of the differences of 5 repeated samples. Box 1 lead total variance was 148 ppm and antimony variance was 2.7 ppm, with a *p*-value of 0.001. Box 2 lead total variance was 54 ppm and antimony was 51 ppm, with a *p*-value of 7.2138 × 10^–05^. Box 3 lead total variance was 36 ppm, whereas antimony total variance was 89 ppm. The *p*-value for this box was 8.2424 × 10^–09^. Box 4 lead total variance was 120 ppm and antimony was 5 ppm. The *p*-value for this box was 0.06, which shows that these values do not reflect a pattern that is applicable to the entire population of this box. In other words, different bullets from this box may not yield the same values as the 5 tested. Box 5 total lead variance was 715 ppm and antimony was 16 ppm. The *p*-value for box 5 was 4.1948 × 10^–14^. Box 6 total lead variance was 315 ppm and antimony was 0.4 ppm. The *p*-value for box 6 was 0.06. Box 7 lead total variance was 3033 ppm and antimony was 1.01 ppm.

The *p*-value for box 7 was 0.5. Box 8 lead total variance was 95 ppm and antimony was 0.7 ppm. The *p*-value for box 8 was 0.0002. Box 9 lead total variance was 343 ppm and antimony was 1.5 ppm. The *p*-value for box 9 was 0.8. Box 10 lead total variance was 234 ppm and antimony was 1.5 ppm. The *p*-value for box 10 was 1.9773 × 10^–06^.

Comparison of averages between boxes were analyzed with the Tukey–Kramer Multiple Comparisons test. Absolute difference values of lead and antimony established difference or no difference between brands relative to each mean. Absolute difference between ratios was also determined with the Tukey–Kramer Multiple Comparisons test shown in Table 4. Lead concentrations of boxes 1–10 showed HSD above 28.8. Antimony concentrations of boxes 1–10 showed HSD above 7.2. Lastly, ratios of boxes 1–10 showed HSD above 7.3.

**Table 4:** Tukey–Kramer Multiple Comparisons: Lead/Antimony Absolute Difference: RWS (Box 1), BPR (Box 2), Armscor Precision (Box 3), Aguila Original (Box 4), CCI Stinger (Box 5), Aguila Super Caliber (Box 6), Aguila Rifle Match (Box 7), Federal Premium (Box 8), La Norma USA (Box 9), and HVHP Seller & Bellot (Box 10)

Tukey–Kramer multiple comparisons: lead/antimony absolute difference										
abs diff	box 1	box 2	box 3	box 4	box 5	box 6	box 7	box 8	box 9	box 10
box 1	N/A	8.6	10.5	2.0	1.3	3.3	10.7	27.1	6.2	0.06
box 2	8.6	N/A	1.9	10.6	9.9	11.9	19.3	35.7	14.8	8.6
box 3	10.5	1.9	N/A	12.5	11.8	13.8	21.2	37.6	16.7	10.5
box 4	2.0	10.6	12.5	N/A	0.64	1.4	8.7	25.1	4.3	2.0
box 5	1.3	9.9	11.8	0.64	N/A	2.0	9.4	25.8	4.9	1.4
box 6	3.3	11.9	13.8	1.4	2.0	N/A	7.4	23.8	2.9	3.4
box 7	10.7	19.3	21.2	8.7	9.4	7.4	N/A	16.4	4.5	10.8
box 8	27.1	35.7	37.6	25.1	25.8	23.8	16.4	N/A	20.9	27.1
box 9	6.2	14.8	16.7	4.3	4.9	2.9	4.5	20.9	N/A	6.3
box 10	0.06	8.6	10.5	2.0	1.4	3.4	10.8	27.1	6.3	N/A

## 4 Discussion

Studies have shown that ammunition can vary between lots in respect to concentration of the elements detected. The manufacturer may not always produce the bullets with exact concentrations of alloy each time, so there is potential variability between lots if metals are added with inconsistent volumes or homogeneity is not achieved in production. This is useful when the values obtained by AAS match the tested lot numbers from the manufacturer of the bullet based on the range of antimony and lead percentages along with the various trace metals or impurities that are indicative of specific brands. For example, the Koons & Grant study conducted by the FBI in 2002 examined the variation of lot numbers using various samples from 2 smelters belonging to a company that produces lead alloys for bullets. Seven metals were detected (Sb, Cu, Ag, Bi, Sn, As, Cd) in the 19 lots analyzed, which is indicative that this is signatory for this manufacturer when the range of values obtained from each element correlate to this composition in future analysis.^10^

A correlation is shown between the results of the Koons & Grant study and this analysis of 10 ammunition brands via AAS. Variation within each brand of bullets could be due to the inconsistency in production that is explained in research. More specifically, the lead concentrations were observed to have far greater ranges than those of antimony. It could be that the method used to pour the alloy into molds was not sufficient for equal distribution of metal throughout the entirety of lots. The material used in the alloy mix could also have scrap metal, which includes variable metal components and concentrations. However, these possible limitations in bullet production do not hinder analysis due to variation in brand-specific metals. For example, the 7 metals detected may not all consistently be present in every manufacturer’s formulations that will lead to distinguishability. Variation between lots can also assist investigators to identify the exact lot in which the ammunition originated, which would not be possible in the absence of inner-lot variation. While inner-lot variation does pose an issue, this can be alleviated with increased sample runs and quality control cross examination.

There are many scenarios in which AAS analysis of projectiles can be used in crime scene investigations given lot variation and distinguishability between manufacturers. Projectile scratches from contact with any substrate such as building materials, cars, furniture, human tissue, etc. will provide an elemental characterization when deposits are extracted from the sample’s surface and analyzed with AAS. Elemental concentrations will be compared to a reference database and a potential brand match will be determined. It is possible for manufacturer composition reporting for each lot to become routine with implementation of a database for examiners to generate a match or elimination for a particular brand and lot number. With results obtained through simple and time efficient sample runs via AAS and database comparison, it will be possible for investigators to ascertain the lot number of the ammunition, location of purchase, and by whom in a single day.

In cases where bullets, casings, and or weapon(s) are on scene, AAS analysis can be used in conjunction with microscopy to add evidentiary value. This technique can also be useful for bullet samples that are collected in small fragments that would otherwise result in inconclusive findings.

## 5 Limitations

There were possible limitations to this study as each sample, including the 5 sample repeats, showed variation. Issues such as variability of the AAS flame and possible heterogenicity of the mixture components unevenly dispersed at the time of injection could have influenced the reported elemental concentrations. Due to these differences in repeated sample runs, it has been determined that these limitations were the most influential in this study. This issue was not alleviated with a change of dissolution time but can be resolved with increased nitric acid concentration during digestion or different solvent based on target metal analysis. Additionally, 6 point-calibration curves for each element prior to all sample runs were obtained equal to or above a linear regression of 0.995 to reduce variation from mechanical error that could have been the limiting factor in this study.

## 6 Conclusion

Elemental analysis via AAS can provide increased probative value to expert’s statements in several aspects. For example, when bullets or cartridges are found on scene but not the firearm, the establishment of a database with known values and specific elemental ratios correlating to the type of bullet and manufacturer can be used for comparison when AAS analysis is conducted on the collected sample. Once a link between the manufacturer is observed, the lot number’s elemental values can be compared with the manufacture’s results recorded from quality control procedures, allowing the investigator to eventually determine when the lot number was sold, the location at which the lot was purchased, and by whom. When the projectiles and the firearm are unobtainable at the crime scene, the elemental database of manufactures can be referenced after AAS analysis of striations or impact samples. Values obtained from this instrumentation can be corroborated with a certain brand and type of ammunition for firearm identification. Additionally, it is possible that material from unjacketed bullets can be extracted and analyzed from the inner wall of a casing and can provide further evidence when the casings are found on scene.

With increased scientific basis in ballistics analysis, the expert’s testimony will no longer rely upon subjective observation only. AAS bullet analysis has the potential to revolutionize crime scene investigation by providing additional, objective evidence that is not influenced by current, subjective protocols. Surface scratches and debris deposits from projectile contact can now be evaluated to determine bullet composition and the firearm involved in the crime, which in turn, can lead investigators to potential suspects. It is imperative that current methods for firearm identification be advanced due to the thousands of violent crimes committed per year, thousands of cold cases, and wrongful convictions based on evidence that may not be suitable for admissibility by Daubert standards. Acquisition of these values and development of an elemental database promises great value to forensic ballistics investigations and opportunity for expansions of this methodology that will address additional concerns in this field. Possibilities of these future endeavors includes the provision of AAS data for inner wall extractions from unjacketed bullet material to further enhance the ability of investigators to perform analyses even in the absence of ballistics evidence on the scene.
